# *Arbuscular mycorrhizal* Fungi and Changes in Primary and Secondary Metabolites

**DOI:** 10.3390/plants11172183

**Published:** 2022-08-23

**Authors:** Mostafa Amani Machiani, Abdollah Javanmard, Reyhaneh Habibi Machiani, Amir Sadeghpour

**Affiliations:** 1 Department of Plant Production and Genetics, Faculty of Agriculture, University of Maragheh, P.O. Box 55136-553, Maragheh 83111-55181, Iran; 2 Crop, Soil and Environment Program, School of Agricultural Sciences, Southern Illinois, University of Carbondale, College of Science, Carbondale, IL 62901, USA

**Keywords:** *Arbuscular mycorrhizal* fungi, alkaloids, essential oil, secondary metabolites, terpenes

## Abstract

Medicinal and aromatic plants (MAPs) are able to synthesize a diverse group of secondary metabolites (SMs) such as terpenoids or terpenes, steroids, phenolics, and alkaloids with a broad range of therapeutic and pharmacological potentials. Extensive use of MAPs in various industries makes it important to re-evaluate their research, development, production, and use. In intensive agricultural systems, increasing plant productivity is highly dependent on the application of chemical inputs. Extreme use of chemical or synthetic fertilizers, especially higher doses of N fertilization, decrease the yield of bioactive compounds in MAPs. The plant–soil microbial interaction is an eco-friendly strategy to decrease the demand of chemical fertilizers. *Arbuscular mycorrhizal* fungi (AMF), belongs to *phylum Glomeromycota*, can form mutualistic symbiotic associations with more than 80% of plant species. The AMF–plant symbiotic association, in addition to increasing nutrient and water uptake, reprograms the metabolic pathways of plants and changes the concentration of primary and secondary metabolites of medicinal and aromatic plants. The major findings reported that inoculation of AMF with MAPs enhanced secondary metabolites directly by increasing nutrient and water uptake and also improving photosynthesis capacity or indirectly by stimulating SMs’ biosynthetic pathways through changes in phytohormonal concentrations and production of signaling molecules. Overall, the AMF-MAPs symbiotic association can be used as new eco-friendly technologies in sustainable agricultural systems for improving the quantity and quality of MAPs.

## 1. Introduction

In recent years, about 5000 plant species were investigated for chemical compositions with pharmacological and biological activities. Among these, medicinal and aromatic plants (MAPs) are able to synthesize a diverse group of secondary metabolites (SMs) such as terpenoids or terpenes, steroids, phenolics, and alkaloids, with a broad range of therapeutic and pharmacological potentials, including anticancer, antioxidant, antitumor, antidiabetic, antiviral, antimicrobial, anti-inflammatory, antidepressant, hepatoprotective, antithrombotic, immune protective, cardiovascular improver, memory enhancer, anti-Parkinson’s, anti-AIDS, anti-Alzheimer’s, and anti-cognitive impairment effects [1,2]. Moreover, SMs play a key role in plant performance as signaling molecules, chemical defense mechanism and adaptation, pollination and seed dispersal, protection from herbivores, predators, pathogens, and allelopathic agents. Additionally, plant SMs are extensively used in food industries as preservatives or anti-browning agents and also coloring, flavoring, or texturizing agents [3].

Extensive use of MAPs in various industries including pharmaceutical, cosmetic, food, etc., makes it important to re-evaluate their research, development, production, and use. In intensive agricultural systems, increasing plant productivity is highly dependent on the application of chemical inputs such as chemical fertilizers, pesticides, and herbicides [4]. The massive application of chemical inputs in these farming systems, in addition to human health hazards, cause negative impacts on the environmental ecosystems, including water and air pollution, acidification of the soil, mineral depletion of the soil, soil erosion, etc. [5]. Moreover, excessive use of chemical fertilizer, especially higher doses of N fertilization, decreases the yield of bioactive compounds in MAPs [6,7]. Therefore, due to the possibility of negative impacts of using various chemical inputs on the quantity and quality of active ingredients of MAPs, the need to use eco-friendly strategies is necessary.

Recently, plant–soil microbial interaction has become an eco-friendly strategy to reduce the demand of chemical fertilizers in agricultural systems. *Arbuscular mycorrhizal* fungi (AMF), belonging to the *Glomeromycota phylum* of fungi, can form mutualistic symbiotic associations with flowering plants, bryophytes, and ferns [8]. The AMF–host plant symbiosis is the most ancient plant-mutualistic association that was reported over 400 million years ago [9]. The positive impacts of AMF symbiosis with plant roots are multiple and variable. The most important effect of AMF on plants is increasing the availability of macro- (especially P) and micronutrient (especially Fe and Zn) uptake due to their extensive hyphal network [10,11,12,13]. Fall et al. [14] noted that AMF can acquire P from less bioavailable P-minerals through enhancing soil biological and enzyme activity and also acidification by releasing H^+^. Also, AMF can enhance water uptake through improving the root hydraulic conductivity as a result of larger surface area of the mycelium [15,16,17]. Another symbiotic benefit of AMF and host plants is improving the photosynthetic rate by regulating the chloroplast enzyme activity, reducing chlorophyll decomposition rate, and promoting chlorophyll synthesis [8,18]. Interestingly, AMF can increase the tolerance of plants in stressful conditions (drought, salinity, temperature herbivory, metals) through augmentation of antioxidant defense systems [19,20]. Amani Machiani et al. [10] noted that inoculation of AMF with soybean plants enhanced the antioxidant enzyme activity, including superoxide dismutase (SOD), ascorbate peroxidase (APX), and guaiacol peroxidases (GPX), which improved plant performance in drought stress conditions. Moreover, AMF can reprogram the metabolic pathways of plants, resulting in changes in the primary (such as sugars, organic acids, amino acids, etc.) and also secondary metabolites (terpenoids, alkaloids, flavonoids, etc.). The productivity and accessibility of the above-mentioned compounds play an important role in plant performance, especially in stressful conditions. It is worth noting that the changes in the primary and secondary metabolites affected by AMF depend on the plant species, AMF species, environmental conditions, and the interaction of the three previously mentioned factors. In this review, we evaluate the effects of AMF on the primary and secondary metabolite changes in host plants.

## 2. Primary metabolites

### 2.1. Sugars

The AMF colonization rate depends on the C-sink strength of host plant roots. In the AMF–plant symbiotic association, sugar is known as an important regulator. In order to sustain the symbiosis life cycle, about 20% of the total fixed photosynthetic carbons from plants transfer to AMF in the form of sugars and lipids [21,22]. Therefore, the higher C assimilation rate in plant roots causes a stronger symbiotic association. At the beginning of the AMF colonization, the higher concentration of hexoses (such as glucose and fructose) was observed in the roots [23]. However, at later stages of plant growth, especially two months after colonization, the higher sugar content was observed in non-mycorrhizal roots as a result of further hexose metabolization by mycorrhizal sink roots [24]. In addition to increasing the sugar content in roots, AMF can increase the concentration of soluble sugars in the plant’s leaves. For example, the total sugars and starch content in *Heptacodium miconioides* Rehder leaves were enhanced after AMF inoculation [25]. It is worth noting that the AMF effects are highly functional under low nutrient availability, especially in P deficiency conditions [23]. After the assimilation of hexoses by AMF, the hexoses convert to trehalose (a fungal-specific sugar) and glycogen in the intra-radical mycelium, which leads to an increase in the protection of host plant roots against abiotic stresses [26]. Table 1 represents the effects of AMF species on the sugar content in different plant species. 

### 2.2. Amino Acids

Amino acids, as the main structures of protein compounds and enzymes, can help to alleviate abiotic stress like drought or salinity. In stressful conditions, plants accumulate varied amino acids for osmotic process regulation, modulating stomatal opening, ion transport, etc. [32]. Also, amino acids have an impact on the biosynthesis and activity of enzymes that affect plant performance in face of stress conditions [33]. It has been reported that the rate of amino acids uptake from soil enhanced with AMF symbiotic association [34]. Moreover, AMF spores are able to synthesize amino acids through nitrogen uptake from soils [23]. It is worth noting that the changes (increase or decrease) in different amino acids in plants are varied and depend on the AMF and plant species, as well as the environmental conditions. Metwally et al. [35] noted the concentration of essential amino acids, such as phenylalanine, isoleucine, leucine, histidine, lysine, methionine, threonine, and valine; as well as the non-essential amino acids such as glycine, arginine, aspartic, serine, glutamic, cysteine, alanine, tyrosine, and proline, in onion enhanced after AMF inoculation. The content of aspartic and glutamic acids was significantly enhanced in *Anchusa officinalis* L. roots through colonization with *Rhizophagus irregularis* [36]. Concomitantly, Rivero et al. [37] noted that the inoculation of AMF with tomato decreased the concentration of phenylalanine, tyrosine, tryptophan, alanine, and leucine, while the concentration of asparagine, and glutamic, aspartic, and pyroglutamic acid increased.

### 2.3. Organic Acids

Generally, inoculation of AMF with host plant roots can increase the content of organic acids in plants and soils and improve plant performance through two different ways: 

(i) The AMF–plant symbiotic association has a positive effect on the synthesis of organic acids, such as the tricarboxylic acid (TCA) cycle. The TCA cycle and availability of other intermediate compounds, including malic acid, fumaric acid, and citric acid affect the synthesis of ATP and cellular respiration, which helps to increase plant performance and improves plant tolerance in the face of stress conditions. Shtark et al. [38] reported that the higher accumulation of organic acids, such as aconitate, lactate, and malonate in pea leaves was observed at the later stages of AMF development. Colonization with *Rhizophagus irregularis* enhanced the content of gluconic acid, threonic acid, malic acid, and phenylacetic acid in *Anchusa officinalis* L. shoots under semi-hydroponic cultivation [36].

(ii) The AMF can release organic acids into the soil, which leads to an increase in the solubility of minerals and could improve their uptake rate by plants [39,40]. MA et al. [41] exhibited that the application of different species of AMF increased the secretion of various organic acids from maize roots. These authors noted that the inoculation of *Funneliformis mosseae* enhanced the exudation of p-coumaric, p-hydroxybenzoic, and caffeic acid; the inoculation of *Claroideoglomus etunicatum* increased the exudation of syringic acid; and *Rhizophagus aggreatus* inoculation promoted the exudation of chlorogenic and succinic acid in maize roots. It is worth noting that the increase of all mentioned organic acids promotes nutrient accumulation in various organs of this plant.

### 2.4. Fatty Acids

Plants synthesize a huge variety of fatty acids that divide into saturated fatty acids such as palmitic, stearic, and lauric acid, and unsaturated fatty acids such as oleic, linoleic, linolenic, etc. The ratio of unsaturated fatty acids to saturated fatty acids determines the oil quality. The oil quality extracted from oil-seed crops was improved by increasing the ratio of unsaturated fatty acids to saturated fatty acids. Previous studies showed that AMF have positive impacts on the oil content and oil quality through increasing the availability of precursor compounds involved in fatty acid biosynthesis. Amani Machiani et al. [10] reported that the inoculation of *Funneliformis mosseae* with soybean plants enhanced the oil content and its quality in comparison with non-mycorrhizal plants. The authors noted that the concentration of oleic and linoleic acids of soybeans increased by 6.8% and 7.4% after AMF colonization. Also, inoculation of *Funneliformis mosseae* + *Rhizophagus irregularis* with black cumin seedlings enhanced the content of oleic and linoleic acids by 2.3% and 4.6%, respectively, when compared with non-mycorrhizal plants [42].

## 3. Secondary Metabolites

Today, more than 80% of people in developing countries use herbal drugs for their primary healthcare [2,43]. The therapeutic effects of medicinal and aromatic plants are attributed to the existence of bioactive compositions belonging to SMs, such as terpenoids, flavonoids, phenolics, alkaloids, glycosides, tannins, etc. [1]. Among the mentioned compounds, terpenes or terpenoids, alkaloids, and flavonoids are the largest group of secondary metabolites [44]. In addition to the positive effects of the AMF–plant symbiotic association on the nutrient and water uptake, increasing productivity and quality of crops, improving soil fertility, and improving tolerance to stress conditions, they also have positive effects on the SM quantity and quality of medicinal and aromatic plants.

### 3.1. Terpenoids

Terpenoids are the main bioactive compounds of essential oils (EOs). EOs or essences are concentrated and volatile lipophilic mixtures of secondary metabolites. Many thousands of EO compounds, such as monoterpenes (like limonene, linalool, geraniol, nerol, terpineol, etc.) and sesquiterpenes (like bisabolene, germacrene, humulene, cadinene, etc.), belong to a vast majority of the terpene family. EOs are used in many industries, such as the food and pharmaceutical industry, due to their characteristic flavor and fragrance properties, as well as other biological activities. Several studies have reported that the AMF inoculation improved EO quantity and quality (Table 2). For example, Amani Machiani et al. [11] noted that the inoculation of *Funneliformis mosseae* with thyme seedlings improved EO quantity and quality through increasing the main EO constituents, such as thymol, *p*-cymene, and *γ*-terpinene, under drought stress conditions. The content of geranyl acetate, thymol, *p*-cymene, borneol, and trans-caryophyllene in black cumin EO increased by 42.39%, 16.68%, 6.80%, 46.11%, and 26.54% after inoculation with a mixture of *Funneliformis mosseae* + *Rhizophagus irregularis* [42].

### 3.2. Alkaloids

Alkaloids act as defense compounds against pathogens and predators due to their toxicity [50]. The nitrogen-containing organic compounds derived from the decarboxylation of amino acids and divided into seven different groups, including tropane, pyrrolidine, pyrrolizidine, benzylisoquinoline, indolequinoline quinolizidine, and piperidine based on their amino acid precursors [51,52]. Until now, more than 20,000 alkaloid compounds have been discovered, most being isolated from plants—of which 600 are known as bioactive compounds [50]. Numerous previous studies reported the increase of alkaloid content after AMF inoculation. Colchicine, the main alkaloid of *Gloriosa superba* L., increased significantly in different parts of the plant (tuber, aerial shoot, and seeds) after inoculation with different AMF species [53]. The content of trigonelline, a plant alkaloid with therapeutic potential, was enhanced by 80.7% in *Prosopis laevigata* (Willd.) M.C. Johnst roots through inoculation with *Gigaspora rosea* [54]. 

### 3.3. Phenolics

Phenolics are one of the SMs produced in plant tissues for protection against ultraviolet radiation or aggression by pathogens, parasites, and predators [55]. Phenolic compounds isolated from plant sources include flavonoids, phenolic acids, tannins, coumarins, curcuminoids, lignins and lignans, stilbenes, and quinones [56]. In plants, phenolic compounds play an important role in cell wall thickening, osmoregulation, hormone production, fruit flavoring, and fruit protection [57]. Like other secondary metabolites, AMF symbiotic associations with host plants have positive effects on the phenolic compounds. Rashidi et al. [18] reported that the content of phenolic compounds in flowers of *Ipomoea purpurea* L. enhanced by 50%, 55.8%, and 71%, respectively, after colonization with *Funneliformis mosseae*, *Rhizoglomus fasciculatum*, and *Rhizoglomus intraradices*. Furthermore, the authors reported that the colonization of *F. mosseae* and *R. intraradices* increased the flavonoids content in roots of *Solanum nigrum* L. by 34% and 41% in comparison with non-AMF plants. The total phenols content in *Passiflora alata* Curtis was enhanced by 110.75% and 93.85% after colonization with *Acaulospora longula* and *Gigaspora albida*, respectively [58]. Duc et al. [59] noted that the mixture of AMF species (*Septoglomus deserticola*, *Funneliformis mosseae*, *Acaulospora lacunosa*) enhanced the total phenolic contents of *Eclipta prostrata* L. plants by 178.5% after 8 weeks. The tannins content of *Libidibia ferrea* (Mart. ex Tul.) L.P. Queiroz fruits were enhanced by 40% after inoculation with *Acaulospora longula* [60].

### 3.4. Saponins

Saponins, as an important group of plant secondary metabolites, display a variety of biological activities of interest to the pharmaceutical, cosmetic, and food sectors [61]. Previous studies reported the increase of saponins content after AMF colonization with host plants. The saponins content of *Passiflora alata* increased by 157.08% after colonization with *Acaulospora longula* [58]. The glycosylated triterpernoids enhanced sharply in shoots of *Anchusa officinalis* L. plants after inoculation with *Rhizophagus irregularis* [36].

### 3.5. Mechanisms of AMF Symbiosis on the Production of Secondary Metabolites

**Figure 1:**
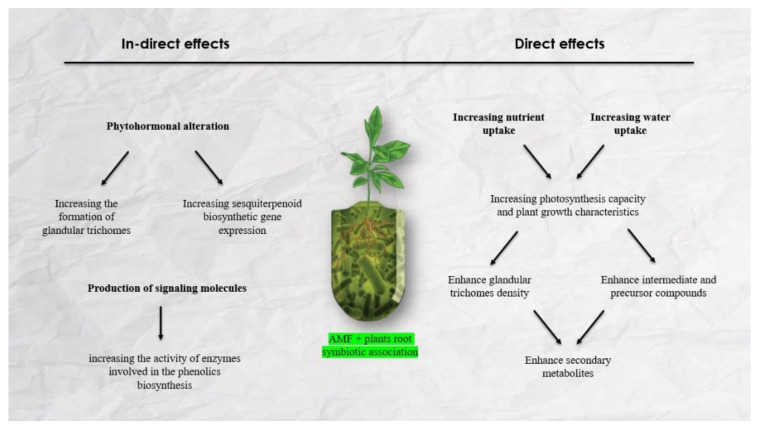
The direct and indirect effects of AMF symbiosis with plant roots on the production of secondary metabolites.

Generally, AMF symbiotic associations with host plant roots can affect plants’ secondary metabolites through two different ways (Figure 1):

(i) Direct effects: In this case, the AMF–plant symbiotic association increases nutrient and water uptake, photosynthetic capacity, and improves the production of SMs through the enhancement of intermediate and precursor compounds [43]. For instance, terpenoids compounds are synthesized in the methyleritrophosphate and mevalonic acid pathways and other secondary metabolites, such as phenolic, flavonoids, and some alkaloids, are synthesized in the phenylpropanoid pathway [23,62]. It seems that the increase of P accessibility through AMF colonization enhanced the precursor compounds, such as NADPH, ATP, acetyl-CoA, pyruvate glyceraldehyde phosphate, erythrose-4-phosphate, and phosphoenolpyruvate, that are required for the biosynthesis of the above-mentioned secondary metabolites [63]. On the other hand, improving nutrient and water uptake enhances plant photosynthetic capacity, which leads to an increase in the development and division of the glandular trichomes, EO channels, and secretory ducts [11]. 

(ii) Indirect effects: In addition to the availability of nutrients, the concentration of secondary metabolites in plants are affected by phytohormonal secretions [64]. In this case, AMF symbiotic associations with host plants change the concentration of phytohormones, including gibberellic acid, cytokinins, and jasmonic acid [65]. It has been reported that jasmonic acid and gibberellic acid enhanced the concentration of terpenoid constituents through increasing the formation of glandular trichomes and sesquiterpenoid biosynthetic gene expression [66]. On the other hand, the signaling molecules among AMF–host plant associations can affect the concentration of SMs in plants. The symbiotic association between *Funneliformis mosseae* and *Trifolium repens* enhances the content of the signaling molecules, including salicylic acid, nitric oxide, and hydrogen peroxide, which lead to the increasing of the activity of enzymes involved in the phenolics biosynthesis [67]. 

## 4. Conclusions

The results of the previous studies showed that the AMF symbiosis association with plant roots act as an active bridge between the soil and plant and improve plants’ primary and secondary metabolites through increasing nutrient and water uptake, enhancing photosynthetic capacity, changing the concentration of phytohormones, and producing signaling molecules. However, the information regarding the effectiveness of different AMF species on plants and their primary and secondary metabolites under various environmental conditions is scant. Gathering information about the effects of new AMF species on the mechanisms involved in the formation of primary and secondary metabolites in different environmental conditions could probably be used in developing new eco-friendly technologies in sustainable agricultural systems.

## Figures and Tables

**Table 1:** Changes of sugar content in different plant species influenced by AMF inoculation.

Sugars	AMF Species	Plant Organs	Plant Species	Environmental Conditions	Changes	Reference
Total sugars	*Funneliformis mosseae*	Leaves	*Medicago sativa* L.	-	Increase	[27]
Total sugars	*Glomus* sp.	Roots + leaves	*Ipomoea batatas* L.	Drought stress	Increase	[28]
Total sugars	*Rhizophagus intraradices*	Leaves	*Triticum aestivum* L.	Arsenic contaminated soil	Increase	[29]
Glucose	*Glomus versiforme*	Roots	*Poncirus trifoliata* L.	Well-watered	Increase	[30]
Sucrose, glucose	*Glomus versiforme*	Roots	*Poncirus trifoliata* L.	Drought stress	Increase	[30]
Fructose, Sucrose	*Glomus versiforme*	Leaves	*Poncirus trifoliata* L.	Drought stress	Increase	[30]
Trehalose	*Glomus intraradices*	Roots	*Medicago truncatula*	-	Increase	[31]

**Table 2:** Effects of different AMF species on the terpenoids content of medicinal and aromatic plants.

Terpenes	AMF Species	Plant Species	Changes	Reference
*β*-elemene, *β*-caryophyllene, germacrene A, germacrene D	*Rhizophagus intraradices*	*Ocimum tenuiflorum* L.	Increase	[45]
Thymol, *P*-cymene, *γ*-terpinene	*Funneliformis mosseae*	*Thymus vulgaris* L.	Increase	[11]
*β*-caryophyllene, *p*-cymene, geraniol	*Glomus hoi*	*Coriandrum sativum* L.	Increase	[46]
Linalool, menthone, pulegone, verbenol acetate	*Rhizophagus irregularis*	*Satureja Macrostema* (Benth.) Briq.	Increase	[47]
camphor, *α*-humulene, viridiflorol, manool, *α*-thujone, *β*-thujone	*Rhizophagus clarus*	*Salvia officinalis* L.	Increase	[48]
Linalyl acetate	*Rhizophagus intraradices*, *Trichoderma harzianum*, *Bacillus subtilis*	*Lavandula angustifolia* Mill.	Increase	[49]

## Data Availability

Not applicable.
